# Serine/threonine Kinases Play Important Roles in Regulating Polyunsaturated Fatty Acid Biosynthesis in *Synechocystis* sp. PCC6803

**DOI:** 10.3389/fbioe.2021.618969

**Published:** 2021-01-21

**Authors:** Gao Chen, Yuelei Cao, Huairong Zhong, Xiaodong Wang, Yanle Li, Xiaoyan Cui, Xiaoyuan Lu, Xiangdong Bi, Meixue Dai

**Affiliations:** ^1^School of Life Sciences, Shandong Normal University, Jinan, China; ^2^Biotechnology Research Center, Shandong Academy of Agricultural Sciences, Shandong Provincial Key Laboratory of Genetic Improvement, Ecology and Physiology of Crops, Jinan, China; ^3^Key Laboratory of Aquatic-Ecology and Aquaculture of Tianjin, College of Fishery, Tianjin Agricultural University, Tianjin, China

**Keywords:** microalgae, serine/threonine kinase system, polyunsaturated fatty acids, biosynthesis, *Synechocystis* sp. PCC6803

## Abstract

Serine/threonine kinases (STKs) play important roles in prokaryotic cellular functions such as growth, differentiation, and secondary metabolism. When the external environment changes, prokaryotes rely on signal transduction systems, including STKs that quickly sense these changes and alter gene expression to induce the appropriate metabolic changes. In this study, we examined the roles of the STK genes *spkD* and *spkG* in fatty acid biosynthesis in the unicellular cyanobacterium *Synechocystis* sp. PCC6803, using targeted gene knockout. The linoleic acid (C18: 2), γ-linolenic acid (C18: 3n6), α-linolenic acid (C18: 3n3), and stearidonic acid (C18: 4) levels were significantly lower in *spkD* and *spkG* gene knockout mutants than in the wild type at a culture temperature of 30°C and a light intensity of 40 μmol⋅m^–2^⋅s^–1^. The expression levels of fatty acid desaturases and STK genes differed between the *spkD* and *spkG* gene knockout mutants. These observations suggest that *spkD* and *spkG* may directly or indirectly affect the fatty acid composition in *Synechocystis* sp. PCC6803 by regulating the expression of fatty acid desaturases genes. Therefore, the STK genes *spkD* and *spkG* play important roles in polyunsaturated fatty acid biosynthesis in *Synechocystis* sp. PCC6803. These findings could facilitate the development of cyanobacteria germplasm resources that yield high levels of fatty acids. In addition, they provide a theoretical basis for the genetic engineering of cyanobacteria with improved yields of secondary metabolites and increased economic benefits.

## Introduction

Polyunsaturated fatty acids (PUFAs) are fatty acids with multiple unsaturated bonds (any number of carbon atoms). Long chain PUFAs has multiple unsaturated bonds and more than 18 carbon atoms in the fatty acid chain. PUFAs are divided into multiple categories based on the position where the unsaturated bond starts relative to the methyl end of the fatty acid carbon chain, using the ω (omega) numbering system. ω-3 and ω-6 PUFAs play important roles in various organisms (Mayra et al., 2020). In addition to having important physiological functions, these PUFAs, including linoleic acid (C18: 2), α-linolenic acid (C18: 3n3), γ-linolenic acid (C18: 3n6), stearidonic acid (C18: 4), arachidonic acid (C20: 4n6), eicosapentaenoic acid (C20: 5), and docosahexaenoic acid (C22: 6), have high nutritional and medicinal value. PUFAs are critical to the function and structure of the nervous system, especially in children. Thus, the demand for PUFAs is increasing. The large-scale industrialization of PUFAs would provide a solution to the PUFA supply and demand (Jan et al., 2010).

PUFAs have traditionally been derived from oils obtained from deep-sea fish and shellfish (Pauly et al., 2005; Hibbeln et al., 2006). However, factors such as seasonal restrictions, increased environmental pollution, overfishing, and residual fishy odor during the purification of PUFAs from fish oil have limited the production of PUFAs (Drexler et al., 2003). Cyanobacteria have been genetically engineered to produce PUFAs that do not have the shortcomings of traditional PUFAs sources (Gong and Miao, 2019; Somayeh et al., 2019). These prokaryotes use sunlight and carbon dioxide to produce a variety of valuable metabolites. Cyanobacteria have beneficial characteristics that make them ideal for large-scale cultivation, including a rapid growth rate and high metabolic yield (Jan et al., 2010). The single-cell cyanobacterium *Synechocystis* sp. PCC6803, a facultative autotrophic organism that uses light energy, is considered a model organism to study PUFA biosynthesis (Guedes et al., 2011; Sara et al., 2015).

Serine/threonine kinases (STKs) play important roles in the growth, differentiation, and secondary metabolism of prokaryotic cells (Rajagopal et al., 2003). When the external environment changes, prokaryotes such as cyanobacteria rely on signal transduction systems that include STKs to quickly perceive changes, and through the precise regulation of gene expression, alter their metabolic processes accordingly. The mechanism employed by STKs in cyanobacteria is similar to that in eukaryotes. After receiving the signal, STKs become activated through phosphorylation of their serine and threonine residues, and cooperate with other signal molecules. For example, when exposed to ultraviolet radiation, osmotic stress, heat stress, salt stress, or cold stress, STK proteins are activated and phosphorylate their downstream molecules, ultimately allowing these external signals to be transmitted to the nucleus (Zhang et al., 2007). The precise regulation of gene expression (Hasegawa et al., 2000) leads to changes in metabolite levels, allowing the cyanobacterium to withstand various types of adversity (Liu et al., 2015; Piyoosh et al., 2019).

*Synechocystis* is the first cyanobacterium whose entire genome has been sequenced. Based on its genome sequence, *Synechocystis* contains seven STK genes, including *spkA* (*sll1574*), *spkB* (*slr1697*), *spkC* (*slr0599*), *spkD* (*sll0776*), *spkE* (*slr1443*), *spkF* (*slr1225*), and *spkG* (*slr0152*) (Zhou et al., 2011). *spkA* and *spkB* of *Synechocystis* sp. PCC6803 contribute to cell movement, *spkE* encodes a protein lacking kinase activity, *spkC*, *spkF*, and *spkK* have been found to be involved in the phosphorylation of the small molecular chaperone protein GroES (Zorina et al., 2011) and *PknD* of *Anabaena* sp. PCC7120 is involved in regulating nitrogen metabolism (Zhang and Libs, 1998; Zhou et al., 2015). Therefore, we first constructed *spkD* and *spkG* knockout mutant strains, and identified a potential connection between these genes and PUFAs.

The main function of STKs is signal transduction. Several STK genes are involved in cell growth and cell survival, but their roles in unsaturated fatty acid biosynthesis is remaining unknown. We previously performed qRT-PCR of STK genes in *Synechocystis* sp. PCC6803 at 2, 4, and 6 days of culture, finding that *spkD* and *spkG* were expressed at significantly higher levels in strains with high levels of PUFAs compared to the wild type when grown at a temperature of 30°C and a light intensity of 40 μmol⋅m^–2^⋅s^–1^. In addition, there have been related reports showing that *spkG* plays an important role in STKs (Liang et al., 2011), and we obtained two knockout mutants, *spkG* and *spkD*. These findings suggest that *spkD* and *spkG* play important roles in unsaturated fatty acid biosynthesis in *Synechocystis* sp. PCC6803.

Most studies of the unsaturated fatty acid biosynthesis pathway conducted in *Synechocystis* to date have aimed to identify gene functions through analyses of mutants with metabolic deficiencies. However, our knowledge of the genes that function in *Synechocystis* fatty acid metabolism is incomplete. STKs have a variety of physiological functions in the cyanobacteria *Synechocystis* sp. PCC6803 and *Anabaena* sp. PCC7120, such as biological movement, osmotic pressure regulation, and cell survival. For example, the STK genes *spkA* and *spkB* are thought to function in cell movement (Kamei et al., 2001); *spkH* is thought to help maintain osmotic pressure in the cell (Kalyanee et al., 2004); and *spkD* affects cell survival (Laurent et al., 2008). However, the roles of other STK genes are still being explored, and the roles of STKs in PUFA biosynthesis in cyanobacteria are currently unknown.

Through the screening of STKs genes and the construction of plasmids, we obtained two mutants: *spkD* and *spkG*. Preliminary experimental results showed that PUFA accumulation in *spkD* and *spkG* deletion mutants was significantly lower than that in the wild type. Therefore, we carried out subsequent experiments using these mutants.

In this study, we further studied the role of *spkD* and *spkG* in PUFA biosynthesis in *Synechocystis* sp. PCC6803. We constructed *spkD* and *spkG* deletion mutant strains of *Synechocystis* sp. PCC6803 (*spkD*- and *spkG*-) and measured the expression levels of fatty acid desaturases and STK genes in the mutants at various points throughout the lifecycle. Fatty acid desaturases catalyze the dehydrogenation of carrier-bound fatty acids to form double bonds in the fatty acid chain. Fatty acid desaturases play an important role in the metabolism of fatty acids and in the maintenance of the correct structure and biological function of the membrane in the organism. Therefore, fatty acid desaturases were analyzed in our experiment. The contents of four unsaturated fatty acids were lower in the *spkD* and *spkG* deletion mutants than in the wild type, whereas the expression levels of fatty acid desaturases genes were higher in the mutants, indicating that *spkD* and *spkG* play important roles in PUFA biosynthesis in *Synechocystis* sp. PCC6803. This study lays the foundation for further investigating the effects of other STK genes on fatty acid biosynthesis.

## Results

### Inactivation of *spkD* and *spkG* in *Synechocystis* sp. PCC6803

**FIGURE 1:**
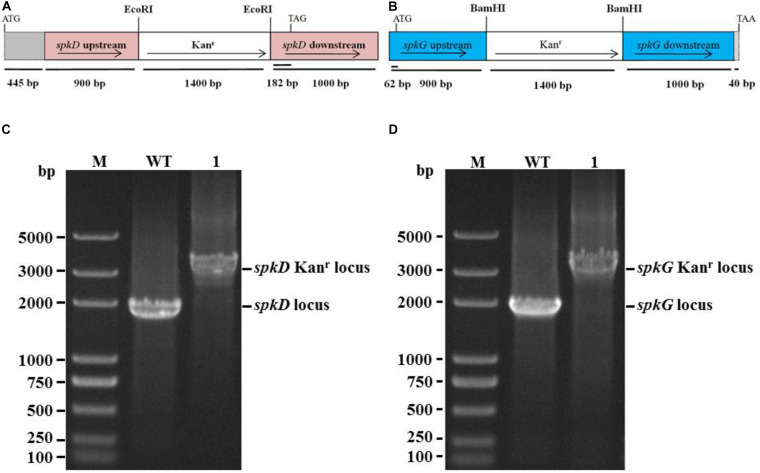
Construction of Synechocystis spkD and spkG gene knockout vectors and PCR detection of mutant strains. **(A)** Diagram of the *spkD* gene-directed knockout vector. The gray region (445 bp) shows the unamplified area in the ORF of *spkD*; **(B)** Diagram of the *spkG* gene-directed knockout vector. The gray region (40 bp) shows the unamplified area in the ORF of *spkG*; **(C)** PCR detection of *spkD* gene-directed knockout, M: *Trans* 2K plus DNA Marker, WT: *spkD* gene in wild type *Synechocystis*, Lane 1: *spkD* gene knockout mutant; **(D)** PCR detection of *spkG* gene knockout, M: *Trans* 2K plus DNA Marker, WT: *spkG* gene in wild type *Synechocystis*; Lane 1: *spkG* gene knockout mutant.

We constructed *spkD* and *spkG* deletion plasmids (Figures 1A,B) by replacing these genes with a kanamycin resistance cassette and *spkD* and *spkG* were separated into 0.9 and 1.0 kb fragments, respectively. These plasmids were used to transform into *Synechocystis* sp. PCC6803 to obtain the *spkD* Kan^r^ locus and *spkG* Kan^r^ locus mutants, here after referred to as *spkD*-and *spkG*-, respectively. To verify that *spkD*/*spkG* were successfully knocked out, we conducted PCR analysis using primer pair 1 (*spkD*-F and *spkD*-R) and primer pair 2 (*spkG*-F and *spkG*-R; Table 1). Whereas primer pairs 1 and 2 amplified 1.9 kb fragments from wild type genomic DNA, they amplified 3.3 kb fragments from the mutant genomic DNA. All amplification products were of the predicted size (Figures 1C,D). PCR is confirmed that all copies of *spkD*/*spkG* in the *Synechocystis* sp. 6,803 genome were replaced by the deletion cassette. Thus, we successfully knocked out the *spkD* and *spkG* genes in *Synechocystis*, and obtained two deletion mutant strains.

**TABLE 1:** Primer sequences used in this study.

Primer	Sequence (5′–3′)
*spkD*-F	ACTTACCCGTTCTGATTGA
*spkD*-R	TAACCATTGATAAGCAGAT
*spkG*-F	AGACTTTCTCTATTGCCTC
*spkG*-R	GGACCCAAATCCAGAAGAC
*rnpB*-F	GTGAGGACAGTGCCACAGAA
*rnpB*-R	GGCAGGAAAAAGACCAACCT
16S rRNA-F	AGCGTCCGTAGGTGGTTATG
16S rRNA-R	CTACGCATTTCACCGCTACA
*spkD*-RT-F	TGAGCCAGCACTTCCA
*spkD*-RT-R	CCACAATAATCCCAATAAGA
*spkG*-RT-F	CGACATTTATGCTGTGGGTA
*spkG*-RT-R	GGGCAAGTAAGGGAGGA
*spkA*-RT-F	TGTAGCGGATGCTGGAC
*spkA*-RT-R	ACTCAACACGGATATGGAA
*spkB*-RT-F	CAAATTGATTCGGTCCTCT
*spkB*-RT-R	TTCCCAGTCCATCTCCC
*spkC*-RT-F	GCCACCAAGGTTTACACTC
*spkC*-RT-R	CCGCCAATCACTAGCAGTA
*spkF*-RT-F	TCGCCATGACCAGATTC
*spkF*-RT-R	CACCCAACGCACTTCC
*d15D*-RT-F	TCGCCTCAAACAAAGC
*d15D*-RT-R	AATCGGATAGAAGAACCAG
*d6D*-RT-F	GCCATTGATGACGAGTG
*d6D*-RT-R	TAGCCAGCGATAGTTAGAG
*d9D*-RT-F	GGCATTGGCATTACTTT
*d9D*-RT-R	CCTTATTAGAATCGTGGG
*d12D*-RT-F	TGGACAGGGACAGCCTTAAC
*d12D*-RT-R	TTTTGTTGGTGTGGAGGTGA

### Growth Characteristics of Wild Type and Mutant Strains

**FIGURE 2:**
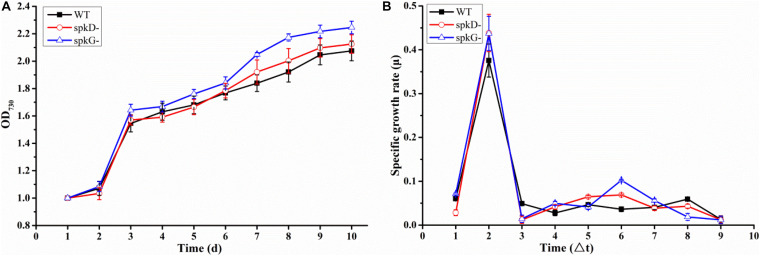
Growth curves and specific growth rates of mutant and wild type cultures under normal light conditions **(A)** Growth curves of wild type *Synechocystis* sp. PCC6803 and two knockout mutants; **(B)** Specific growth rates of wild type *Synechocystis* sp. PCC6803 and two knockout mutants. WT represents wild type; *spkD* represents *spkD* knockout mutant; and *spkG* represents *spkG* knockout mutant. The experiment was carried out under a normal light intensity of 40 μmol⋅m^–2^⋅s^–1^. △t is the length of the time interval in days. Values are means ± SD (bars) of three independent experiments conducted on different days. The absence of a bar indicates that the SD falls within the symbol.

To determine whether the knockout of *spkD* and *spkG* genes would affect the normal growth of *Synechocystis*, we incubated the cultures for 10 days under normal light intensity (40 μmol⋅m^–2^⋅s^–1^) and measured the OD_730_ value once daily. Based on these OD_730_ values, we generated growth curves of the wild type and two mutant strains. The growth patterns of all three *Synechocystis* strains were roughly similar (Figure 2A). The OD_730_ values of the knockout mutants were slightly higher than that of the wild type under the same conditions; the final OD_730_ value of the *spkG* knockout mutant was the highest. Nevertheless, the overall differences among the three strains were small, indicating that the algal cell density and growth rates were approximately the same.

We generated a standard curve of *Synechocystis* density and OD_730_ (y = 32,798x + 6,249, *R*^2^ = 0.9896). Using this growth curve, the density of *Synechocystis* under different OD values could be determined. We conducted a statistical analysis of the growth rates of the two mutant strains and the wild type under normal light conditions (Figure 2B). The growth rates of all three cyanobacterial strains peaked on the second day of culture, began to increase slightly on the fourth day, and reached a second small peak on the sixth day. However, after this stage of culture, the growth rate began to decline until it reached the lowest value and became stable. Overall, there was no significant difference in growth rates between the mutant and wild type strains.

### Effects of Knocking Out *spkD* and *spkG* Under Normal Light Conditions on STK Gene Expression in *Synechocystis*

**FIGURE 3:**
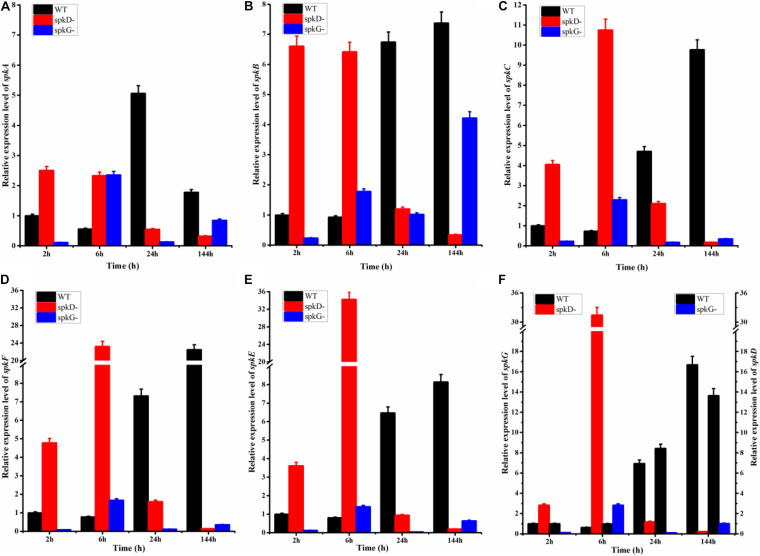
Changes in serine/threonine kinase (STK) gene expression in wild type and mutant strains detected after different periods of exposure to normal light. WT represents wild type *Synechocystis* sp. PCC6803; *spkD*- represents the *spkD* knockout mutant; *spkG*- represents the *spkG* knockout mutant. The experiment was carried out under a normal light intensity of 40 μmol⋅m^–2^⋅s^–1^. **(A–E)** show the relative expression levels of *spkA*, *spkB*, *spkC*, *spkF*, and *spkE*, respectively, in the wild type and two mutant strains. **(F)** Relative expression levels of *spkG* in the wild type and *spkD*-. The data correspond to the left vertical axis. The black bars represent the wild type. *Synechocystis* sp. PCC6803, and the red bars represent the mutation that knocked out *spkD*. The right vertical axis shows the relative expression levels of *spkD* in the wild type and *spkG*-, and the black bar represents the wild type. The blue bar represents the mutation that knocked out *spkG*. Values are means ± SD (bars) of three independent experiments conducted on different days. The absence of a bar indicates that the SD falls within the symbol.

To examine the effect of *spkD* and *spkG* gene knockout on the expression of other STK genes, we conducted follow-up experiments investigating differences in the expression levels of these related genes in wild type and mutant *Synechocystis* sp. PCC6803 under normal light conditions. The STK-related genes were generally expressed at lower levels in the *spkD* knockout mutant than in the wild type (Figure 3). Except for *spkA* transcript levels at 24 h of light treatment, the differences in gene expression patterns between the mutant and wild type strains were substantial, with a maximum difference in expression observed at approximately 6 h of light treatment, especially in the case of *spkE*. Knockout of *spkD* had the greatest effect on *spkE* expression, pointing to a possible association between these genes. In addition, the STK-related genes were expressed at higher levels in the mutant than in the wild type *Synechocystis* sp. PCC6803 before 6 h of light treatment, suggesting that *spkD* might be involved in regulating the expression of some inhibitors during the early stages of STK biosynthesis.

We also examined the expression of STK-related genes in the *spkG* knockout mutant under the same conditions. Under normal light conditions, the difference in *spkA* and *spkB* gene expression in the *spkG* knockout mutant vs. the wild type was greatest at 24 h, and the expression of *spkC*, *spkD*, *spkE*, and *spkF* increased overtime (Figure 3). The difference in gene expression also gradually increased over time. Since the knockout of *spkG* strongly affected *spkC*, *spkD*, *spkE*, and *spkF* expression, we speculate that *spkG* is associated with the expression of these genes in *Synechocystis* sp. PCC6803.

### Effects of Knocking Out *spkD* and *spkG* on the Expression of Fatty Acid Desaturases Genes in *Synechocystis*

Fatty acid desaturases is a key enzyme in the PUFA biosynthesis pathway, catalyzing the formation of double bonds at specific locations on the fatty acid chain. There are four types of fatty acid desaturasess in cyanobacteria: delta 6 fatty acid desaturases, delta 9 fatty acid desaturases, delta 12 fatty acid desaturases and delta 15 fatty acid desaturases. Referred to, respectively, as *d6D*, *d9D*, *d12D*, and *d15D*, these fatty acid desaturases differ depending on where they form double bonds in fatty acids.

**FIGURE 4:**
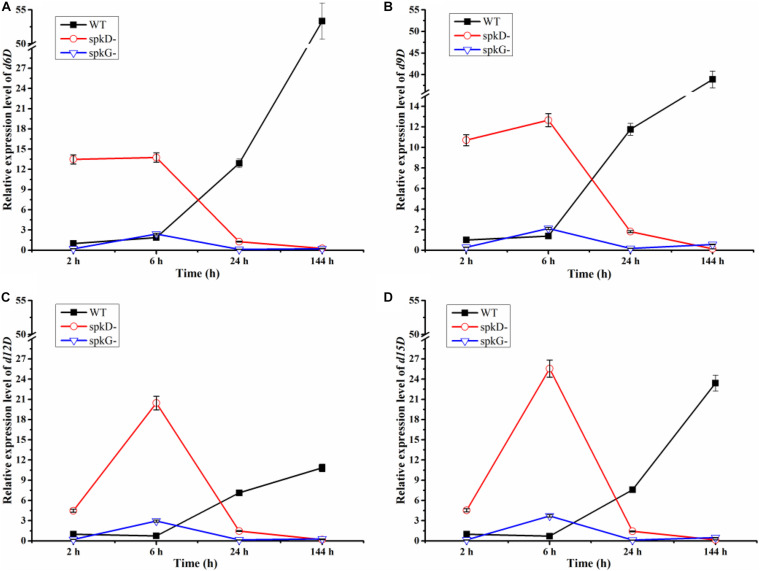
Changes in fatty acid desaturases gene expression in the wild type, *spkD* and *spkG* knockout mutants under normal light conditions. WT represents wild type *Synechocystis* sp. PCC6803; *spkD*- represents the *spkD* knockout mutant; *spkG*- represents the *spkG* knockout mutant. The experiment was carried out under a normal light intensity of 40 μmol⋅m^–2^⋅s^–1^. (**A–D** show the relative expression levels of the *d6D, d9D, d12D, and d15D* fatty acid desaturases genes, respectively, in wild type *Synechocystis* sp. PCC6803 and the two knockout mutants) Values are means ± SD (bars) of three independent experiments conducted on different days. The absence of a bar indicates that the SD falls within the symbol.

Using qRT-PCR, we compared the expression patterns of the four fatty acid desaturases genes in the wild type and mutant strains at different time points under normal light conditions. The expression of the fatty acid desaturases related genes tended to decrease over time in both mutant strains, but to increase in the wild type. These genes were expressed at higher levels in the *spkD* knockout mutant than in the *spkG* knockout mutant. Under normal light conditions, desaturases are expressed at higher levels in the *spkD* deletion mutants than in the wild type during the early stages of growth, with peak expression at 6 h in the mutant. Because the expression level of the wild type was low in the early stages of culture, the difference between the expression level of the wild type and that of the two mutant strains reached a maximum at 6 h. However, this difference decreased over time, and the genes were ultimately expressed at higher levels in the wild type (Figure 4). These results indicate that *spkD* and *spkG* are strongly associated with the expression of fatty acid desaturases genes in *Synechocystis* sp. PCC6803 (Figure 4).

In addition, we compared the expression levels of the four fatty acid desaturases genes in the two mutant strains. In the *spkG* knockout mutant, the expression of the four fatty acid desaturases genes was maintained at relatively low levels. The expression patterns of all four genes in the *spkD* knockout mutants were opposite to those of the wild type. These results indicate that knockout of *spkG* affects the expression of fatty acid desaturases genes and that this gene has a greater influence on fatty acid desaturases gene expression than *spkD*.

We speculate that, after 6 h of light exposure, the expression level of fatty acid enzymes of the mutant strains transiently increased compared with the wild type. After 24 h of light treatment, the expression level of these genes decreased in the mutants, to levels that were lower than in wild-type *Synechocystis* sp. PCC6803. After 144 h of light exposure, the knockout of *spkD* and *spkG* in the mutant strain further inhibited the expression of fatty acid enzyme genes, leading to further reductions in their expression in the mutants. Thus, deletion of *spkD* and *spkG* decreased the expression of the four genes (encoding *d6D*, *d9D*, *d12D*, and *d15D*).

### Changes in Fatty Acid Contents in the *spkD* and *spkG* Knockout Mutants

**FIGURE 5:**
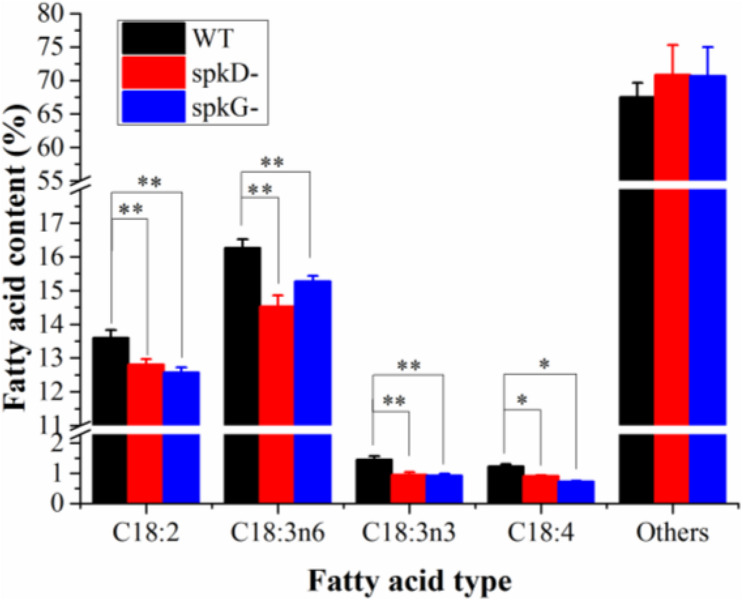
Fatty acid composition and content in *spkD* and *spkG* knockout mutants. Data are the mean values of three experiments; the cultivation temperature was 30°C and the light intensity was 40 μmol⋅m^–2^⋅s^–1^; WT: *Synechocystis* sp. PCC6803; *spkD*-: *spkD* knockout mutant strain; *spkG*-: *spkG* knockout mutant strain. Values are means ± SD (bars) of three independent experiments conducted on different days. The absence of a bar indicates that the SD falls within the symbol. Asterisks mark statistically (*t*-test) significant differences from the WT value (*^∗∗^p* < 0.01; *^∗^p* < 0.05).

To characterize differences in fatty acid content between the mutant and wild type, we analyzed the fatty acid contents of *Synechocystis* sp. PCC6803 cultured at 30°C and a light intensity of 40 μmol⋅m^–2^⋅s^–1^ by gas chromatography. There was little difference in total fatty acid content in the wild type and mutant strains (Figure 5). However, the contents of C18: 2, C18: 3n6, C18: 3n3, and C18: 4 were slightly lower in the mutants than in the wild type. The contents of other fatty acids were lower in the wild type than in the mutants, and the contents of these fatty acids in the two mutants were similar. The C18: 3n3 and C18: 4 contents were significantly lower in the *spkG* than in the *spkD* knockout mutant.

## Discussion

Genetic engineering to modify metabolic pathways represents an efficient, convenient method for increasing the yields and types of unsaturated fatty acids produced by an organism. The fatty acid biosynthetic pathways in plants and cyanobacteria have been well studied. *Synechocystis* sp. PCC6803 is a single-cell cyanobacterium, making it an ideal choice for studying fatty acid biosynthetic pathways whose potential as a tool for the commercial production of biofuels and fatty acids has been explored (Ruiz-López et al., 2012). For example, the delta 6 and delta 15 fatty acid desaturases genes were transferred into *Synechocystis* sp. PCC6803 to produce unsaturated fatty acids (Chen et al., 2014).

Cyanobacteria are the earliest photosynthetic autotrophic organisms. During the long evolutionary process, cyanobacteria developed an efficient adaptive system to cope with the constant changes in the external environment. For example, a complex signal transduction network system in *Synechocystis* sp. PCC6803 allows it to adapt to a variety of environments (Lei et al., 2014). The prokaryotic cyanobacteria are dominated by a binary signal transduction system. In 1991, eukaryotic protein kinases including STKs were also identified in the prokaryotic organism *Myxococcus* and shown to play major roles in prokaryotic cells (Zorina et al., 2014).

STKs contain multiple genes, and two mutant strains, *spkD* and *spkG*, were successfully obtained by knockout. The genes knocked out in the *spkD* and *spkG* strains have been well characterized, but the association between these genes and fatty acids remains to be explored. Based on this, we studied these two genes.

Here, to verify the roles of STKs in unsaturated fatty acid biosynthesis in *Synechocystis* sp. PCC6803, we knocked out these genes in this cyanobacterium and obtained gene knockout mutants. Gas chromatography analysis revealed that the contents of C18: 2, C18: 3n6, C18: 3n3, and C18: 4 were significantly lower in the two mutant strains than in the wild type. The C18: 3n3 and C18: 4 contents were significantly lower in the *spkG* than in the *spkD* knockout mutant, whereas the contents of other unsaturated fatty acids such as C18: 2 and C18: 3n6 in the two mutants were similar.

To verify this finding, we extracted RNA from the cultures and performed qRT-PCR to analyze fatty acid-related gene expression levels. Fatty acid desaturases genes were expressed at higher levels in the two mutant strains vs. the wild type, and the expression levels of these genes in the *spkD* and *spkG* knockout mutants significantly differed at different time points and under different light intensities. These findings suggest that *spkD* and *spkG* play different roles in unsaturated fatty acid biosynthesis.

We also analyzed the changes in fatty acid desaturases gene expression in the mutants and wild type at different time points under normal light intensity. The expression levels of these genes in the wild type and mutant strains were significantly different. Under normal light conditions (40 μmol⋅m^–2^⋅s^–1^), the fatty acid desaturases genes were expressed at higher levels in the *spkD*- mutant than in the *spkG*- mutant prior to 144 h of cultivation. Unlike the wild type, these genes were expressed at their highest levels in the mutants at 6 h of light treatment, with the most obvious differences detected in the *spkD* knockout mutant. After the 6 h time point, the expression levels of these genes in the wild type continued to increase. These results indicate that knockout of *spkD* and *spkG* indeed affects the relative fatty acid desaturases contents of *Synechocystis* sp. PCC6803, indirectly demonstrating that the two genes are closely related to fatty acid biosynthesis.

Subsequently, we detected the changes in expression levels of other STK genes and established that *spkD* and *spkG* influence the expression patterns of these genes as well, as the knockout of these genes significantly altered the expression levels of other STK genes. Our findings suggest that in *Synechocystis* sp. PCC6803, *spkD* is most closely associated with *spkE* expression and *spkG* is somewhat associated with *spkC*, *spkD*, *spkE*, and *spkF* expression.

In addition, with the extension of light time, in contrast to wild type *Synechocystis*, the expression of other STKs genes in the *spkD* knockout mutants initially increased, before decreasing. Perhaps *spkD* is related to the pre-expression phase of these genes, a process that might involve some inhibitory factors or other genes. Compared to the *spkD* knockout mutant, other STK genes were significantly down regulated in the *spkG* knockout mutant, indicating that *spkG* is more closely associated with the expression of other STK genes than is *spkD*. The expression levels of the STK genes of these two knockout mutants peaked after approximately 6 h of light exposure. The importance of this time point will be the subject of subsequent experiments.

As we had expected, in the subsequent determination of fatty acid content, we also noticed that several fatty acids were significantly higher in the wild type than in the two knockout mutant strains. This difference has to do with fatty acid desaturases and coincides with the results of STKs obtained previously, further illustrating that *spkD* and *spkG* are associated with fatty acid metabolism and are worth further examination.

A previous related study indicated that *sll0776* (*spkD*) is part of the gene cluster (*sll0775*, *sll0776*, *sll0777*, and *sll0778*). However, the intergenic region of this gene cluster could not be amplified by qRT-PCR, indicating that *sll0776* was not organized in an operon with the adjacent genes, and the protein sequences encoded by the other genes had low similarity with the corresponding proteins in the database. Therefore, we chose *spkD* gene as the research object. *spkD* is not thought to be an essential gene, but may be associated with the TCA cycle. Deletion of *spkD* maybe affects cyanobacterial growth, and the growth can be recovered by adding specific metabolites of HCO_3_^–^ or TCA cycle as auxiliary elements (Laurent et al., 2008). In our research, the STK-related genes were expressed at higher levels in the *spkD* deletion mutant strains in the first 6 h, and gradually decreased in the later stages. We speculated that this may be due to a lack of a bit substances related to growth (Figure 3). Another study revealed that certain cyanobacterial genes are expressed soon after exposure to light, and their expression gradually decreases or even disappears at later stages (He et al., 2001). This may also explain why *spkA*, *spkB*, *spkC*, *spkF*, and *spkE* are expressed at relatively high levels suddenly after exposure to light, however, the specific mechanism remains to be elucidated.

Recent studies of *spkG* (*slr0152*) also identified a relationship between spkG kinase and fatty acids. The *slr0144*–*slr0152* gene cluster encodes a protein that was recently annotated as Ferredoxin 5 (Fd5), which appears to be a phosphoprotein. SpkG kinase is involved in the phosphorylation of Ferredoxin 5 (Fd5) (Angeleri et al., 2018). Our study confirms that *spkG* influences the contents of fatty acids. Furthermore, knockout of *spkG* (Δ*slr0152*) will not have an obvious effect on the expression of genes that function downstream of genes in the cluster. We are planning to study other genes in the gene cluster to further explore the connection between *spkG* and fatty acid metabolism. As *slr0151* and *spkG* have similar functions, we speculate that *spkG* kinase and *slr0151* protein may have complementary functions, which would result in the loss of *spkD* inhibiting cyanobacterial growth to a greater extent than the loss of *spkG*. This would also explain why other STKs and fatty acid desaturases are altered greater in *spkD* deletion mutants than in *spkG* deletion mutants (Figures 3, 4).

In this study, we successfully constructed *spkD* and *spkG* gene knockout mutants in *Synechocystis* sp. PCC6803 via insertional inactivation to study the effects of these genes on fatty acid biosynthesis. Under normal light conditions, the expression levels of fatty acid desaturases genes were significantly higher in the wild type than in the two knockout mutants. Knockout of *spkD* and *spkG* also affected the expression of other STK-related genes to different extents, indicating that these genes play different roles in fatty acid biosynthesis. In addition, the contents of several major fatty acids were lower in the mutant strains than in the wild type. Our results indicate that STKs affect fatty acid biosynthesis, and *spkD* and *spkG* directly or indirectly participate in the regulation of STK gene expression. In addition, the knockout of these two genes affected fatty acid biosynthesis. However, how specific genes cooperate with each other to regulate fatty acid biosynthesis mechanisms remains to be determined. We plan to conduct more in-depth research to improve the biosynthesis of fatty acids in cyanobacteria. This study lays the foundation for further improving the fatty acid biosynthesis pathway in cyanobacteria and for the efficient production of PUFAs.

## Materials and Methods

### Strains and Growth Conditions

The cyanobacterium *Synechocystis* sp. PCC6803 was obtained from the Freshwater Algae Culture Collection of the Institute of Hydrobiology, Chinese Academy of Sciences. *Synechocystis* sp. PCC6803 was cultivated in BG-11 medium at 30°C (Chen et al., 2014). For solid BG-11 medium, 1.5% (w/v) Difco Bacto-agar (Becton Dickinson, Sparks, MD, United States), 0.3% (w/v) sodium thiosulfate, and 10 mM TES 2- [(2-hydroxy-1, 1-bis (hydroxylmethyl) ethyl) amino] ethanesulfonic acid pH 8.2 were added to BG-11 medium. The culture was bubbled with air under a light intensity of 40 μmol⋅m^–2^⋅s^–1^ (Teruo and Kaplan, 2003). Transformed strains were selected by adding 50 μg/mL kanamycin (Dingguo Company, Beijing, China) to both liquid and solid BG-11 medium. The mutant and wild type in the logarithmic growth phase were added to the liquid medium without kanamycin. Under a light intensity of 40 μmol⋅m^–2^⋅s^–1^, 30°C, after waiting for a period of growth (about 3–5 days), the OD_730_ value was uniformly adjusted to 1.0. Samples were collected and measured at the same time each day. Cell density was determined by measuring the optical density (OD) of the suspension at 730 nm (OD_730_) with a spectrophotometer (DU-70, Beckman Coulter, Brea, CA, United States).

### Generation of the *spkD* and *spkG* Mutants

A 1.9 kb DNA fragment including the *spkD* coding region (*sll0776*, GenBank: AB046600) was amplified by PCR from genomic DNA of *Synechocystis* sp. PCC6803 using the primer pair *spkD*-F and *spkD*-R (Table 1). A 1.9 kb DNA fragment including the *spkG* (*slr0152*, GenBank: CP028094) coding region was amplified by PCR from genomic DNA of *Synechocystis* sp. PCC6803 using the primer pair *spkG*-F and *spkG*-R (Table 1). The amplified DNA fragments were cloned separately into the pClone007 simple vector (TSINGKE Biological Technology). In our experiments, the *Eco*RI restriction site was found at position 1,327 bp of the *spkD* gene, and the *Bam*HI restriction site was found at position 866 bp of the *spkG* gene. We designed primers for the two gene sequences and inserted Kan fragments using enzyme digestion. The mutant was constructed by inserting a 1.2 kb kanamycin resistance cassette into the restriction site of the amplified DNA fragment. The *Synechocystis* sp. PCC6803 strain was transformed as described (Kamei et al., 2001). Transformants were selected on standard medium containing 50 μg/mL kanamycin. Complete segregation of the mutant was confirmed by PCR.

### RNA Isolation and cDNA Synthesis

Wild type and transformant cell lines were cultured and harvested during the exponential growth phase, and total RNA was isolated from the samples using Trizol Reagent (Invitrogen, Carlsbad, CA, United States) following the manufacturer’s instructions. First-strand cDNA was synthesized using M-MLV reverse transcriptase and modified oligo (dT) following the manufacturer’s instructions (TaKaRa, Dalian, China).

### Quantitative Reverse-Transcription PCR

Quantitative reverse-transcription PCR (qRT-PCR) of STK gene expression was carried out RNA Bio-Rad iQ5 real-time PCR system. The resulting cDNA molecules were amplified by PCR using the following gene-specific primers: *rnpB*-F and *rnpB*-R to amplify the *rnpB* gene and 16S rRNA-F and 16S rRNA-R to amplify the 16S rRNA gene, which was used as the loading control. Primers were also designed to amplify the following STK genes (Table 1): *spkA* (*spkB*, *spkC*, *spkD*, *spkG*, *spkF*) -RT-F and *spkA* (*spkB*, *spkC*, *spkD*, *spkG*, *spkF*) -RT-R to amplify *spkA* (*spkB*, *spkC*, *spkD*, *spkG*, *spkF*); *d6D* (*d9D*, *d12D*, *d15D*) -RT-F and *d6D* (*d9D*, *d12D*, *d15D*) -RT-R to amplify *d6D* (*d9D*, *d12D*, *d15D*). Reactions were prepared following the manufacturer’s instructions, and qRT-PCR was performed using a Bio-Rad iQ5 system. Each PCR was repeated four times in a total volume of 20 μL containing 2 × SYBR Green I PCR Master Mix (TaKaRa), 100 nM of each primer, and 1 μL diluted (1:20) template cDNA. Reactions were carried out in 96-well optical-grade PCR plates and a matched optical-grade membrane (TaKaRa). The amplification program was as follows: an initial denaturation step of 1 min at 95°C; 42 cycles of 10 s at 95°C, 30 s at 60°C, and 30 s at 72°C; and then an additional cycle of 10 s at 95°C, 30 s at 58°C, and 5 min at 72°C, followed by 10 s at 95°C for melting curve analysis. The data were analyzed using Bio-Rad iQ5 software. The relative expression levels of STK genes at various developmental stages were calculated using the relative 2^–Δ^ ^Δ^ ^Ct^ method (Livak and Schmittgen, 2001); the error bars indicate SD (*n* = 3). Sterile water was used as negative control instead of template in each primer set.

### Culture Conditions

Three *Synechocystis* strains (wild type and mutants) were cultured under normal light conditions (40 μmol⋅m^–2^⋅s^–1^) for 10 days at 30°C. After a brief centrifugation (6,000 × g, 10 min at room temperature), the cyanobacteria were added to BG-11 medium to a cell density of 4.578 × 10^9^ ind./L (individual/liter) and cultured under a normal light intensity of 40 μmol⋅m^–2^⋅s^–1^ for 6 days. All treatments had four replicate flasks, and cyanobacteria cultured continuously under 40 μmol⋅m^–2^⋅s^–1^ served as controls. During the light culture stage, algal growth was estimated each day based on the OD_730_, as measured with a spectrophotometer. A standard curve relating *Synechocystis* cell density to OD_730_ was established using serial dilutions of cyanobacterial culture. Total RNA for subsequent qRT-PCR analysis was isolated from the cyanobacteria at 0, 2, 4, 6, 24, and 144 h.

### Calculation of Specific Growth Rate

The specific growth rate of the cyanobacteria was calculated using the following formula: μ = (lnN_t_–lnN_0_)/△t, where N_0_ is the population cell density at the beginning of the time interval, N_t_ is the cell density at the end of the time interval, and △t is the length of the time interval in days. The growth rate was calculated based on measurements taken from the first 2 days of the experiment onward. Data from the second day was compared with that from the first day, and the rate was calculated by comparison.

### Extraction of Total Fatty Acids From *Synechocystis*

*Synechocystis* cells were collected in 1,000 mL autoclaved flasks, each containing 400 mL of sterile BG-11 medium, and grown for 10 days at a light intensity of 40 μmol⋅m^–2^⋅s^–1^ and a constant temperature of 30°C. When the culture reached OD_730_ = 2.2, the cyanobacterial fluid was collected by centrifugation (4,500 × g, 10 min at room temperature), and the pellet was washed with distilled water and centrifuged again (4,500 × g, 10 min at room temperature). The washing and centrifugation steps were performed three times (Chen et al., 2017). The cultured cyanobacterial cells were harvested by centrifugation (6,000 × g, 15 min at room temperature) and dried under a vacuum. Each 0.2 g sample of dried cyanobacterial powder was placed in a mortar, repeatedly ground in liquid nitrogen, extracted with 7.0 mL of methanol-chloroform (2:1, v: v), and sonicated for 10 min. After the sample was centrifuged at 6,000 × g for 15 min at room temperature, 1.5 mL of methanol-chloroform (2: 1, v: v) was added to the residue. The sample was centrifuged (6,000 × g, 10 min at room temperature) and the organic phase was retained; this step was repeated once. The organic phase extracts were combined in a separatory funnel. After adding 2.5 mL of chloroform and 3.0 mL of sodium chloride solution (1:100, v: v), the sample was mixed well and allowed to stand for layer separation. The lower layer was recovered, and 2.5 mL of chloroform was added to the original upper and middle layers and extracted again. The lower layers were combined; this step was repeated once. The combined lower layers were placed in a fat-lifting bottle, and the solvent was evaporated to constant weight with nitrogen at 50°C (Sheng et al., 2011; Ryckebosch et al., 2012). The total fatty acids and the total weight of the fat-lifting bottle were measured using an electronic scale.

### Analysis of Fatty Acid Composition in *Synechocystis*

The extracted total fat was dissolved in 4 mL of chloroform in a 10 mL test tube with a stopper, combined with 5 mL of 0.04 M potassium hydroxide-methanol solution, and mixed well. The sample was incubated in a 60°C water bath for 60 min for saponification, with oscillation every 10 min during saponification. The saponified sample was removed from the water bath, cooled, combined with 4.0 mL hydrochloric acid-methanol (1:9) solution, and mixed well. Then 20.0 μL of 1.5 mg/mL non-adecanoic acid (C19: 0) was added to the sample as an internal standard. The sample was methylated in a water bath at 60°C for 20 min with shaking every 10 min during the methylation process (Ichihara and Fukubayashi, 2010). After cooling, the methylated sample was combined with 3.0 mL of saturated saline, followed by 1.0 mL of n-hexane, and shaken thoroughly. After letting the sample stand (at room temperature for about 30 min), the n-hexane layer was subjected to chromatographic analysis. The extracted n-hexane was dehydrated with an appropriate amount of anhydrous sodium sulfate and centrifuged at high speed (13,000 × g, 3 min) at room temperature (about 30°C). The supernatant [*Synechocystis* sample fatty acid methyl ester (FAME) eluent] was subjected to gas chromatography (GC) using an Elite-wax column in an ASXL instrument (Perkin-Elmer, Waltham, MA, United States) (Kenichi and Yumeto, 2010). The flame ionization detection temperature was 250°C, and the operating temperature was maintained at 220°C. The samples used in the experiment are from the same batch. The value for each sample was averaged over three experiments.

### Statistical Analysis

Data are expressed as means ± SD (*n* = 3). The data were subjected to a *t*-test to determine significant differences between treatments (*^∗^p* < 0.05; *^∗∗^p* < 0.01).

## Data Availability Statement

The original contributions presented in the study are included in the article/supplementary material, further inquiries can be directed to the corresponding author/s.

## Author Contributions

GC, XB, and MD conceived and designed the experiments. YC, HZ, XW, YL, and XC performed the experiments. GC contributed reagents, materials, and analysis tools. GC, YC, and XW wrote the manuscript. All authors have read and agreed to the published version of the manuscript.

## Conflict of Interest

The authors declare that the research was conducted in the absence of any commercial or financial relationships that could be construed as a potential conflict of interest.
