# Erratum: Serine/threonine Kinases Play Important Roles in Regulating Polyunsaturated Fatty Acid Biosynthesis in *Synechocystis* sp. PCC6803

**DOI:** 10.3389/fbioe.2021.686089

**Published:** 2021-04-19

**Authors:** Frontiers Production Office

**Affiliations:** Frontiers Media SA, Lausanne, Switzerland

**Keywords:** microalgae, serine/threonine kinase system, polyunsaturated fatty acids, biosynthesis, *Synechocystis* sp. PCC6803

Due to a production error, there was a mistake in the caption of **Figure 3** as published. The corrected caption appears below.

**Figure 3**. Changes in serine/threonine kinase (STK) gene expression in wild type and mutant strains detected after different periods of exposure to normal light. WT represents wild type *Synechocystis* sp. PCC6803; *spkD*- represents the *spkD* knockout mutant; *spkG*- represents the *spkG* knockout mutant. The experiment was carried out under a normal light intensity of 40 μmol·m^−2^·s^−1^. **(A–E)** show the relative expression levels of *spkA, spkB, spkC, spkF*, and *spkE*, respectively, in the wild type and two mutant strains. **(F)** Relative expression levels of *spkG* in the wild type and *spkD*-. The data correspond to the left vertical axis. The black bars represent the wild type. *Synechocystis* sp. PCC6803, and the red bars represent the mutation that knocked out *spkD*. The right vertical axis shows the relative expression levels of *spkD* in the wild type and *spkG*-, and the black bar represents the wild type. The blue bar represents the mutation that knocked out *spkG*. Values are means ± SD (bars) of three independent experiments conducted on different days. The absence of a bar indicates that the SD falls within the symbol.

The publisher apologizes for this mistake. The original article has been updated.

